# Synergy of EGFR and AURKA Inhibitors in *KRAS*-mutated Non–small Cell Lung Cancers

**DOI:** 10.1158/2767-9764.CRC-23-0482

**Published:** 2024-05-08

**Authors:** Tetyana Bagnyukova, Brian L. Egleston, Valerii A. Pavlov, Ilya G. Serebriiskii, Erica A. Golemis, Hossein Borghaei

**Affiliations:** 1 Program in Cell Signaling and Metastasis, Fox Chase Cancer Center, Philadelphia, Pennsylvania.; 2 Moscow Institute of Physics and Technology, Dolgoprudny, Moscow Region, Russian Federation.; 3 Kazan Federal University, Kazan, Russian Federation.; 4 Department of Cancer and Cellular Biology, Lewis Katz School of Medicine at Temple University, Philadelphia, Pennsylvania.; 5 Division of Thoracic Medical Oncology, Fox Chase Cancer Center, Philadelphia, Pennsylvania.

## Abstract

The most common oncogenic driver mutations for non–small cell lung cancer (NSCLC) activate EGFR or KRAS. Clinical trials exploring treatments for *EGFR*- or *KRAS*-mutated (*EGFR^mut^* or *KRAS^mut^*) cancers have focused on small-molecule inhibitors targeting the driver mutations. Typically, these inhibitors perform more effectively based on combination with either chemotherapies, or other targeted therapies. For *EGFR^mut^* NSCLC, a combination of inhibitors of EGFR and Aurora-A kinase (AURKA), an oncogene commonly overexpressed in solid tumors, has shown promising activity in clinical trials. Interestingly, a number of recent studies have indicated that EGFR activity supports overall viability of tumors lacking EGFR mutations, and AURKA expression is abundant in *KRAS^mut^* cell lines. In this study, we have evaluated dual inhibition of EGFR and AURKA in *KRAS^mut^* NSCLC models. These data demonstrate synergy between the EGFR inhibitor erlotinib and the AURKA inhibitor alisertib in reducing cell viability and clonogenic capacity *in vitro*, associated with reduced activity of EGFR pathway effectors, accumulation of enhanced aneuploid cell populations, and elevated cell death. Importantly, the erlotinib-alisertib combination also synergistically reduces xenograft growth *in vivo*. Analysis of signaling pathways demonstrated that the combination of erlotinib and alisertib was more effective than single-agent treatments at reducing activity of EGFR and pathway effectors following either brief or extended administration of the drugs. In sum, this study indicates value of inhibiting EGFR in *KRAS^mut^* NSCLC, and suggests the specific value of dual inhibition of AURKA and EGFR in these tumors.

**Significance:**

The introduction of specific KRAS G12C inhibitors to the clinical practice in lung cancer has opened up opportunities that did not exist before. However, G12C alterations are only a subtype of all KRAS mutations observed. Given the high expression of AURKA in KRAS^mut^ NSCLC, our study could point to a potential therapeutic option for this subgroup of patients.

## Introduction

In 2022, there were approximately 237,000 new diagnoses of lung cancer, and 130,000 deaths from this disease, in the United States (1). Although abysmal, these numbers nevertheless reflect an improvement in the treatability of lung cancer, based on increasing knowledge of the biology of this disease, and the development of new therapeutic strategies (2). The most common form of lung cancer, non–small cell lung cancer (NSCLC), falls into two primary subtypes: lung adenocarcinoma (LUAD) and lung squamous cell carcinoma. For the more common subtype, LUAD, the most common driver mutations are activation of EGFR (27%) and KRAS (32%), with a minority of tumors dependent on mutations or translocations activating STK11 (17%), ALK (∼8%), or MET (7%; refs. 2, 3). The development and deployment of targeted therapies for these oncogenic drivers (3, 4), coupled with the growing use of biomarker-guided use of immune checkpoint inhibitors (ICI) targeting PD-L1 and other tumor-protective modulators (5, 6), have together resulted in improved prognosis for many individuals with LUAD. Nevertheless, mortality from NSCLC remains high, and many individuals do not respond to ICIs, emphasizing the need to develop better therapeutic strategies.

KRAS-mutated tumors were for a long time considered a challenging tumor type because of the lack of targeted therapeutics targeting RAS proteins. However, over the past decade, the first effective KRAS-targeting agents were developed as covalent modifiers of the KRAS^G12C^ mutation (7). In lung cancer in Western populations, KRAS^G12C^ mutations are extremely common (nearly 50% of all patients; ref. 8). As KRAS^G12C^ inhibitors such as sotorasib have moved into clinical trials, they have resulted in objective responses and increases in progression-free survival for many patients, including those who had previously been treated with ICIs (9). Nevertheless, as for most targeted inhibitors, tumors typically develop resistance to RAS-targeting agents after an initial period of response (10). Hence, it remains critical to identify strategies to address RAS-mutated tumors.

Interestingly, as data have begun to accumulate on sources of resistance to the KRAS^G12C^ inhibitor sotorasib, resistance has been associated with overexpression of EGFR and other receptor tyrosine kinases (RTK; ref. 11). Antibody and small-molecule inhibitors of EGFR were some of the first approved signaling pathway-targeted inhibitors (12, 13), and are used extensively in tumors bearing EGFR mutations or with EGFR overexpressed, as well as in other contexts in which EGFR signaling contributes to tumor growth and survival. Notably, a study of the use of EGFR inhibitors in colorectal cancer tumors with mutations and sotorasib resistance found that upregulation of EGFR was a common resistance mechanism, and dual targeting of EGFR and KRAS blocked acquisition of resistance (14). Although lung tumors with wild-type (wt) EGFR are typically considered resistant to EGFR inhibitors, in contrast to those bearing *EGFR* mutations (4, 15, 16), data from a number of studies indicate that EGFR inhibition can provide a survival benefit in KRAS-mutated tumors (17–19).

In this study, we focus on the evaluation of combination of inhibitors of Aurora-A kinase (AURKA) and EGFR in the context of NSCLC bearing KRAS mutations. AURKA inhibitors have been explored extensively in clinical trials for over 15 years (20), but have not yet been approved clinically for single-agent use due to insufficient activity. However, a number of features of AURKA have maintained interest in this target. A canonical function of AURKA functions is control of mitotic entry, execution, and conclusion, based on roles at centrosomes and the mitotic spindle both in untransformed cells and in tumors. Hence, use of AURKA inhibitors results in spindle abnormalities that induce DNA damage and aneuploidy, promoting cell arrest and lethality. In addition, AURKA is commonly expressed at much higher levels in many tumors, either because tumors lose *TP53*, a repressor of AURKA expression (21), or because of AURKA amplification, which is common in tumors of epithelial origin (22). Furthermore, overexpressed AURKA directly or indirectly promotes phosphorylation and activity of multiple proteins normally activated by EGFR and its proximal effectors. These include AKT, ERK1/2, and proteins that mediate epithelial–mesenchymal transition, associated with drug resistance and tumor invasion (23–26).

In this context, the combination of inhibitors of AURKA and EGFR has been explored and shown promising activity in several tumor types. Synergy between small-molecule inhibitors of EGFR and AURKA was first identified in a synthetic lethality screen performed in EGFR-overexpressing head and neck squamous cell carcinomas and colorectal cancers (27). Inhibition of AURKA has been shown to overcome resistance to the EGFR inhibitors such as the EGFR-targeting antibody cetuximab (28). Clinical trials in progress are assessing potential combination activity of the AURKA inhibitor alisertib with the EGFR inhibitor osimertinib (e.g., NCT04085315) in metastatic EGFR-mutant lung cancer.

Although a number of studies have now shown value of an EGFR-AURKA combination in EGFR-mutated or -overexpressing cancers, the question of whether this combination may have activity in lung tumors that do not contain EGFR driver mutations has not been addressed. These results suggested that an EGFR-AURKA inhibitor combination may offer value in EGFR-wt NSCLC. In this study, we assessed the effect of combining erlotinib and alisertib in a panel of NSCLC cell models without EGFR mutations. This identified benefit of combining these inhibitors *in vitro* and *in vivo*, in NSCLC models with RAS mutations, suggesting potential clinical value in tumors with these genotypes.

## Materials and Methods

### Cell lines, Compounds, and Antibodies

The lung cancer cell lines A549 (RRID:CVCL_0023), H322M (RRID:CVCL_1557), H460 (RRID:CVCL_0459), and HOP-62 (RRID:CVCL_1285) were obtained from the Fox Chase Cancer Center (FCCC) cell culture facility. H358 (RRID:CVCL_1559) and H2228 (RRID:CVCL_1543) were purchased from ATCC. The H1299 (RRID:CVCL_0060) cell line was provided by Dr. George Simon. The human fibroblast cell line FC1010 was provided by Jerome Freed. Negative *Mycoplasma* testing and short tandem repeat profile to confirm identity were performed for each cell line by IDEXX BioAnalytics in 2018. *AURKA* copy number was evaluated by quantitative PCR with primers and probe sequences as follows: forward primer: 5′-TCTTTTATAGAAATGTGTGGAAGTTCCT-3′; reverse primer: 5′-CAATAAAAAAGTACAGACGCATAAACCA-3′; probe: 5′-FAM-CTGTCCTTAGAAATAACCACTAC-TAMRA-3′. Normalization was done with Taqman sets for *RNAseP* (Thermo Fisher Scientific 4403326) and *ALB* (5′-CATTTATTGGTGTGTCCCCTTG-3′; reverse primer: 5′-ACACCAGTGAAAACAATTTAAGCC-3′; probe: 5′-6fam-CCCAACAGAAGAATTCAGCAGCCGTAAG-bhq1-3′. For each marker, PCR was performed in duplicates at 10 and 2.5 ng. Cycling conditions were 95°C, 15 minutes, followed by 40 (two-step) cycles (95°C, 15 seconds; 60°C, 60 seconds). Ct (cycle threshold) values were converted to quantities (in arbitrary units) using a standard curve established with a calibrator sample. For each sample, the values are average and SD of the data derived from four PCR reactions. Genomic DNA isolated from the HCT116 (RRID:CVCL_0291) and SW480 (RRID:CVCL_0546) cell lines, previously reported to have two and four copies of AURKA, (29) served as controls for *AURKA* gene amplification.

Cancer cell lines were maintained in RPMI1640 medium supplemented with 10% FBS and l-glutamine; for FC1010 cells, 15% FBS was used. Erlotinib was purchased from LC Labs; alisertib (MLN8237) from Takeda Pharmaceuticals. All antibodies used in the study are listed in Supplementary Table S1. Anti-EGFR antibody was from BD Transduction Labs, anti-phospho-SRC^Tyr418^ was from Abcam. All other antibodies were obtained from the Cell Signaling Technology. In some experiments, AURKA activity was measured with a phospho-AURKA (ph^Thr288^AURKA) Whole Lysate Kit from Meso Scale Discovery (MSD). Secondary anti-mouse and anti-rabbit antibodies were from Bio-Rad Laboratories, Inc and from LI-COR Biosciences.

### Drug Sensitivity Assays

Cells were plated in 96-well plates with 1,500–8,000 cells per well and incubated overnight. Drugs were added as 10x stocks, and cell viability was measured in 72 hours using the fluorescence-based CellTiter Blue Cell Viability Assay (Promega; catalog no. G8081). The software CompuSyn was used to determine IC_50_ values for erlotinib and alisertib, and synergy by the Chou-Talalay method (30). Evaluation of IC_50_ curves, together with published studies of effective doses of both drugs in mice (31, 32), allowed approximation of range testing for a potentially effective combination dose ratio.

### Cell Cycle Analysis

Cells were plated in 6-well plates and treated with erlotinib, alisertib, their combination or vehicle. Cells were trypsinized, collected and fixed with 70% ethanol 24 or 72 hours after treatment, then stained with PI/RNase staining buffer (BD Biosciences, 550825) and analyzed using a BD FACSymphony A5 Cell Analyzer (BD Biosciences). Data were analyzed with FlowJo software (FlowJo LLC).

### Clonogenic Assay

A549, H358, H322M, and H460 cells were plated in triplicate in 6-well plates with the density of 250 or 10,000 cells per well with drugs or vehicle control added the next day. Cells were grown for 10 days, and the media replaced every 3 days, then fixed in freshly made solution containing 10% acetic acid and 10% methanol for 10–15 minutes, dried and stained with Crystal violet. The plates were scanned and quantified using NIH ImageJ software.

### Western Blot and Phospho-AURKA Analysis

Protein lysates were prepared using T-PER (for tumors) or M-PER (for cultured cells) protein extraction reagent (Thermo Fisher Scientific Inc.) containing protease and phosphatase inhibitors; for tumor lysates, a 5-fold higher concentration of phosphatase inhibitor was used. Protein concentration was determined using a BSA kit using albumin as a standard (Pierce). Western blotting was performed using standard procedures. Signal intensity was quantified using the NIH ImageJ (RRID:SCR_003070) or Odyssey Imaging System software (LI-COR Biosciences) and normalized to loading control (β-actin, vimentin, or GAPDH). In some cases, to increase signal in limited specimens, an MSD kit was used for measuring phospho-AURKA levels; this is a sandwich immunoassay based on electrochemiluminescence. Samples were incubated in a plate precoated with capture antibody for total AURKA on the electrode surface, then with the detection antibody for ph^T288^-AURKA conjugated with an electrochemiluminescent compound, and signal intensity determined using an MSD imager (MSD) as per manufacturer's instructions. Results were normalized to the protein content in the sample. Total AURKA was assessed by Western blotting.

### Mouse Xenografts

All animal experiments were approved by the FCCC Institutional Animal Care and Use Committee. For animal experiments, erlotinib was formulated in 5% DMSO/dextrose including 1% Tween-80. Alisertib was prepared in a mixture of 10% 2-hydroxypropyl-β-cyclodextrin and 1% sodium bicarbonate. C.B-17*scid* mice ages 6–8 weeks were obtained from the Fox Chase Cancer Center breeding colony; both male and female mice were used. To assess drug efficacy, mice were injected subcutaneously in the flank with 3 × 10^6^ A549 and H358 cells. When tumors reached 100–150 mm^3^, mice were randomized into four treatment groups (*n* = 5–8 mice per group): (i) control (vehicle only), (ii) 10 mg/kg erlotinib, daily, (iii) 10 mg/kg alisertib, twice a day, (iv) erlotinib and alisertib at the indicated doses. The approximate drug dose levels for *in vivo* studies were chosen based on published studies [erlotinib, (31); alisertib (32)]. The drugs were administered orally for 3 weeks, then mice were euthanized, and tumors were collected. Tumor volume was calculated using the formula: tumor volume (mm^3^) = (smallest diameter^2^ × largest diameter)/2.

To assess early signaling events following drug addition, mice were injected in both flanks as noted above with A549 or H358 cells. After tumors reached 150–200 mm^3^, mice were treated with a single dose of erlotinib, alisertib, or both, at the indicated above concentrations, with a control group receiving vehicle only. Mice were euthanized 6 or 24 hours posttreatment as indicated, and tumors were frozen for further processing for Western blotting. All control tumors were collected at 6 hours posttreatment.

### Informatic Analysis

We retrieved three genomic datasets [The Cancer Genome Atlas LUAD PanCancer (33); OncoSG (34); CPTAC (35)], containing RNA and protein expression data, mutation information, and copy-number alterations (CNA), from cBioPortal (https://www.cbioportal.org). Data normality was checked using Anderson–Darling tests. To assess differences in gene expression among specific groups, asymptotic two-sample Kolmogorov–Smirnov tests were used. We used *χ*^2^ test to compare proportions of AURKA CNA in different cohorts. All analyses were executed by V.A. Pavlov and I.G. Serebriiskii using R version 4.0.3 and RStudio (RRID:SCR_000432).

### IHC

Tumors were fixed in 10% phosphate-buffered formaldehyde, dehydrated, and embedded in paraffin. IHC was performed according to standard protocols. Antibodies used for IHC are listed in Supplementary Table S1. Results were quantitated with the Vectra Imaging System (PerkinElmer, Inc.), as in ref. 36.

### Statistical Analysis

We (B.L. Egleston) used generalized linear regression models for hypothesis testing of the expression and growth data. We assumed Gaussian family and identity link or Gamma family and log link for the models based on the distribution of the data examined prior to hypothesis testing. We used interaction terms in the models to test for synergy. Wald tests were used to compare model coefficients.

### Data Availability

The data used for informatic analysis were obtained from cBioPortal (https://www.cbioportal.org). The experimental data generated in this study are available upon request from the corresponding author.

## Results

### Expression Relationships Between AURKA and EGFR in EGFR^mut^ versus KRAS^mut^ NSCLC

**FIGURE 1:**
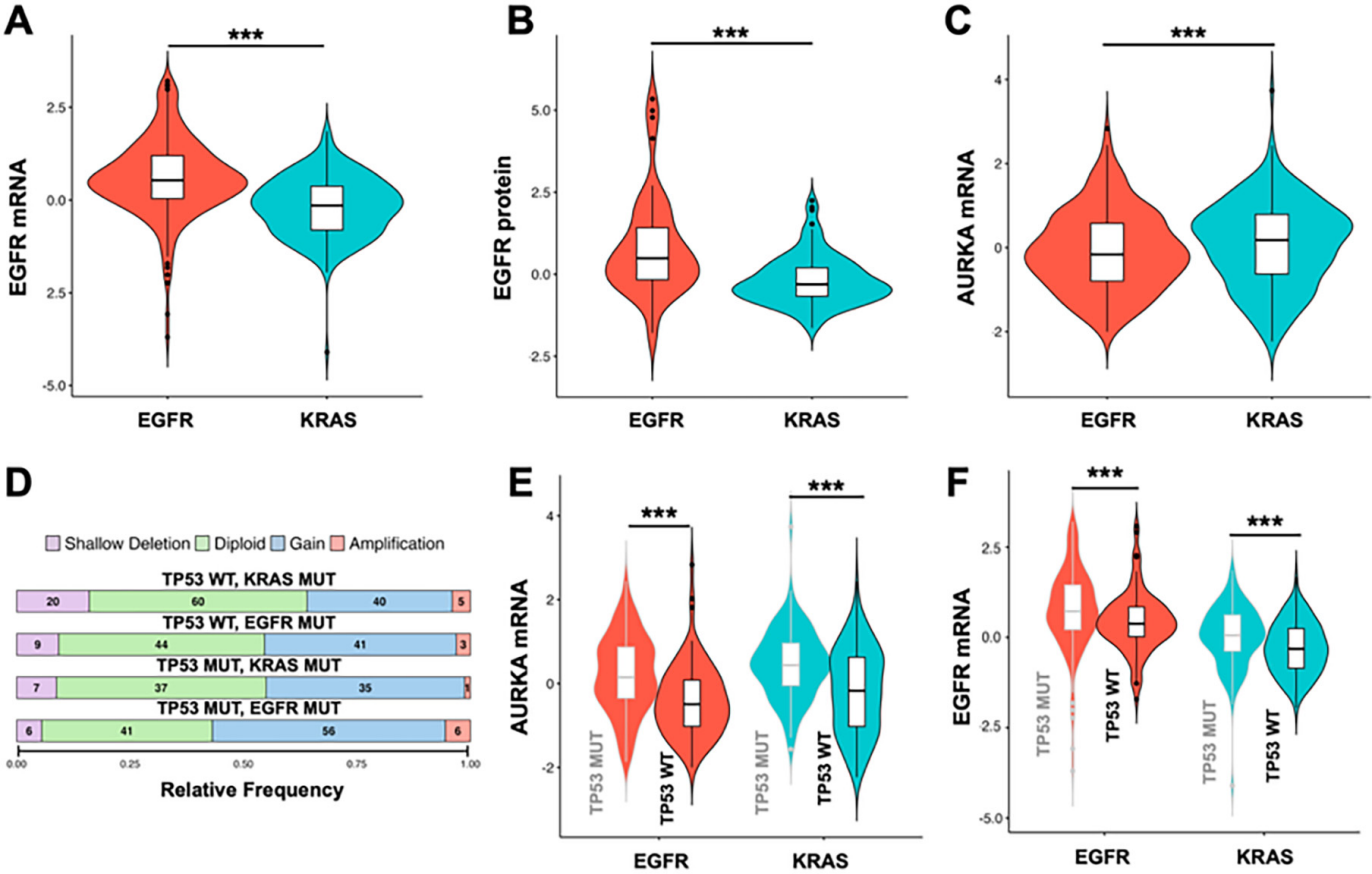
Expression of AURKA and EGFR in NSCLC. **A,** EGFR mRNA expression in KRAS^mut^ versus EGFR^mut^ NSCLC, from a combined set of 789 samples (LUAD PanCancer, OncoSG, and CPTAC studies). Expression data are presented as RSEM z-scored values. **B,** EGFR protein expression in KRAS^mut^ versus EGFR^mut^ in LUAD PanCancer study. Expression data are presented as RPPA z-scored values (356 samples). **C,** AURKA mRNA expression in KRAS^mut^ versus EGFR^mut^ NSCLC, in a combined set of 789 samples (LUAD PanCancer, OncoSG, and CPTAC studies). Expression data are presented as RSEM z-scored values. **D,***AURKA* copy-number changes dependent on mutation status of KRAS, EGFR, and TP53, in a combined set of 411 samples (LUAD PanCancer, OncoSG, and CPTAC studies). **E,** AURKA mRNA expression in KRAS^mut^ versus EGFR^mut^ NSCLC based on TP53 mutation status, in a combined set of 789 samples (LUAD PanCancer, OncoSG, and CPTAC studies). Expression data are presented as RSEM z-scored values. **F,** EGFR mRNA expression in KRAS^mut^ versus EGFR^mut^ NSCLC based on TP53 mutation status in combined set of 789 samples (LUAD PanCancer, OncoSG, and CPTAC studies). Expression data are presented as RSEM z-scored values. See Materials and Methods for details of data retrieval and analysis.

To provide a general context for studies of AURKA and EGFR inhibition in KRAS-mutated (KRAS^mut^) tumors, we first analyzed genomic and transcriptomic data available through cBioportal (37). mRNA and protein levels of EGFR were elevated in EGFR-mutated (EGFR^mut^) versus KRAS^mut^ tumors (median Z-scores 0.53 vs. −0.14 for mRNA, *P*-value = 1.281^e-13^, and 0.48 vs. −0.30 for protein, *P*-value = 3.94^e-05^; Fig. 1A and B; Supplementary Table S2). In contrast, mRNA levels of AURKA were slightly elevated in KRAS^mut^ tumors (median Z-scores −0.16 vs. 0.18; *P* = 0.007; Fig. 1C; Supplementary Table S2). AURKA copy number showed a modest level of gain in approximately 50% of NSCLC, with a frequency unaffected by EGFR, KRAS, or TP53 mutational status (Fig. 1D; Supplementary Table S2). In addition, expression of AURKA mRNA was modestly elevated in tumors bearing *TP53* mutations as reported previously (21), regardless of whether tumors were *EGFR^mut^* or *KRAS^mut^* (Fig. 1E; Supplementary Table S2); Similarly, expression of EGFR mRNA was slightly elevated in *TP53^mut^* tumors [median Z-scores 0.72 (TP53^mut^) vs. 0.38 (TP53^wt^) for *EGFR^mut^*, *P*-value = 0.0012, and 0.05 (*TP53^mut^*) vs. −0.31 (*TP53^wt^*) for *KRAS^mut^* samples, *P*-value = 4.187^e-06^] (Fig. 1F; Supplementary Table S2). Overall, these data indicated abundance *EGFR* and *AURKA* expression in tumors with *KRAS* driver mutations did not differ greatly from abundance in tumors with EGFR driver mutations.

### Synergy Between Erlotinib and Alisertib in Reducing Viability and Clonogenicity of KRAS^mut^ Lung Cancer Cell Lines

**FIGURE 2:**
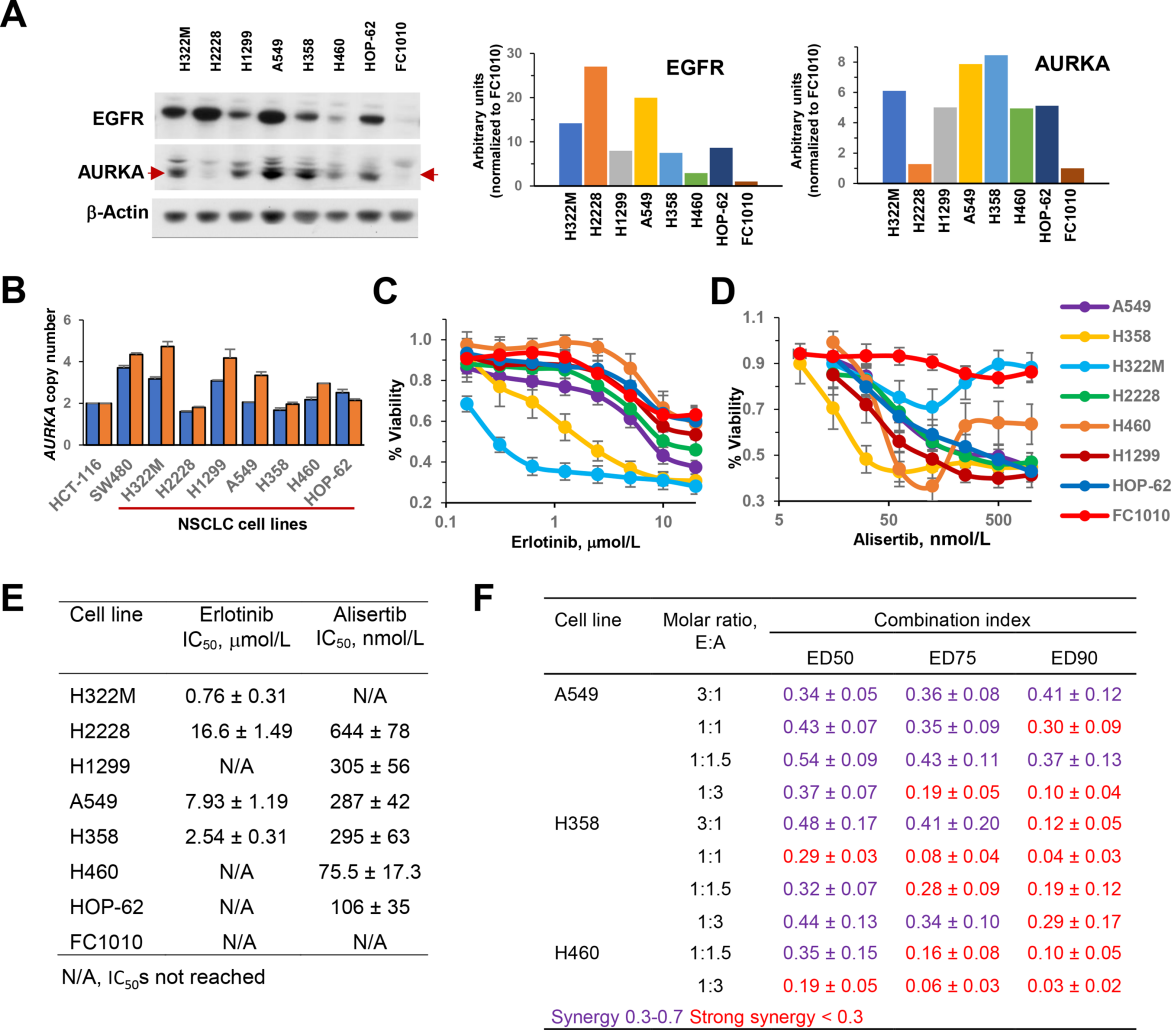
Sensitivity of NSCLC cell lines to erlotinib and alisertib. **A,** Western blot images and quantification of the protein levels of EGFR and AURKA in NSCLC cell lines and the human fibroblast cell line FC1010. Data for NSCLC cell lines are normalized to the levels in FC1010 cells. **B,** Determination of *AURKA* gene copy number in NSCLC cell lines, using the colorectal cancer cell lines HCT116 and SW420 as standards. Normalization to *RNAseP*, blue, or *ALB*, orange. The slopes of the standard curves were −3.64 (*AURKA*), −3,46 (*RNAseP*), −3.49 (*ALB*). Determination of sensitivity of the NSCLC cell lines and FC1010 control to erlotinib (**C**) and alisertib (**D**), with quantified IC_50_ values (**E**), based on CelltiterBlue analysis of viability after growth with indicated drugs for 72 hours. N/A, IC_50_s were not calculated because of low sensitivity to the drug. **F,** Combination indexes for erlotinib and alisertib over a range of molar ratios. Results shown reflect averages from four to five independent experiments, each done in quadruplicate, ± SEM and were calculated using CompuSyn software.

To gauge the efficacy of combining AURKA and EGFR inhibition in KRAS^mut^ lung cancers with wt EGFR, we first confirmed their protein expression in a panel of eight cell lines, including six lung cancer with driver mutations in *KRAS*, *NRAS*, and *EML4-ALK,* one lung cancer (H322M) with a homozygous mutation in *TP53* proposed to have a driver function (38), and a fibroblast control line (FC1010; Table 1; Fig. 2A). Five of the lung cancer models had mutations inactivating the *TP53* tumor suppressor, and quantitative PCR suggested moderate elevation of AURKA copy number in some of the cell models (Table 1; Fig. 2B). Western blot analysis for expression of EGFR and AURKA demonstrated some variation in expression of these proteins across the various cell line models (Fig. 2A); however, in contrast to results for mRNA (Fig. 1), protein levels did not significantly correlate with *TP53* mutation status.

**TABLE 1:** Characteristics of NSCLC cell lines

Characteristics of NSCLC cell lines used in the study					
Cell line	Cell type	EGFR status	KRAS status	Driver mutations	*AURKA* gene copy number^a^
H322M H2228 H1299 A549 H358 H460 HOP-62	Adenocarcinoma Adenocarcinoma Carcinoma Adenocarcinoma Adenocarcinoma Large cell carcinoma Adenocarcinoma	WT WT WT WT WT WT WT	WT WT WT G12S G12C Q61H G12C	*TP53^R248L^* *EML4-ALK, TP53^Q331Ter^* *NRAS^Q61K^, ΔTP53* *KRAS^G12S^* *KRAS^G12C^, ΔTP53* *KRAS^Q61H^, PIK3CA^E545K^* *KRAS^G12C^, TP53^R175L^*	[3.2], [4.7] [1.6], [1.8] [3.1], [4.2] [2.1], [3.3] [1.7], [2.0] [2.2], [3.0] [2.5], [2.1]

^a^Data for copy number are normalized to two separate control genes: [*RNAseP*], [*ALB*].

In measurement of single-agent drug activity (Fig. 2C–E), the cell models showed a range of responses to erlotinib, with IC_50_ varying from 0.76 to 16.6 µmol/L in four lines, and >20 µmol/L in three lines, based on measurement of viability by CellTiterBlue after 3 days of drug exposure (Fig. 2C and E). In contrast, six of seven cell lung cancer lines were sensitive to alisertib, with IC_50_ values ranging from 75.5 to 644 nmol/L (Fig. 2D and E). Interestingly, two lines (H322M and H460) showed a biphasic pattern of response to alisertib, with less growth inhibition observed at the highest doses; this may reflect off-target activity of the drug at higher concentrations (e.g., ref. 39), inhibiting proteins that limit tumor cell growth. Noncancerous FC1010 fibroblasts were not sensitive to either drug. Sensitivity to these drugs in the NSCLC cell lines did not correspond to EGFR or AURKA expression, or to *TP53* mutation status.

Given the high incidence and poor outcomes associated with KRAS^mut^ NSCLC, we further explored the interaction between alisertib and erlotinib in two KRAS^mut^ cell lines, A549 and H358 cells, both of which expressed appreciable levels of AURKA and EGFR, and both of which had significant single-agent response to these drugs (Fig. 2A and E). Over four ratios of drug admixture (erlotinib:alisertib 3:1, 1:1, 1:1.5, 1:3) assessed at multiple combinations, we found synergy or strong synergy at all ratios (Fig. 2F). Interestingly, in further assessment, the KRAS^mut^ H460 cell line also showed synergy to strong synergy to the alisertib-erlotinib drug combination (Fig. 2F), even though this cell model expressed lower levels of EGFR and AURKA than the other models (Fig. 2A), and was resistant to single agent erlotinib (Fig. 2E), suggesting potential broad utility of the drug combination.

**FIGURE 3:**
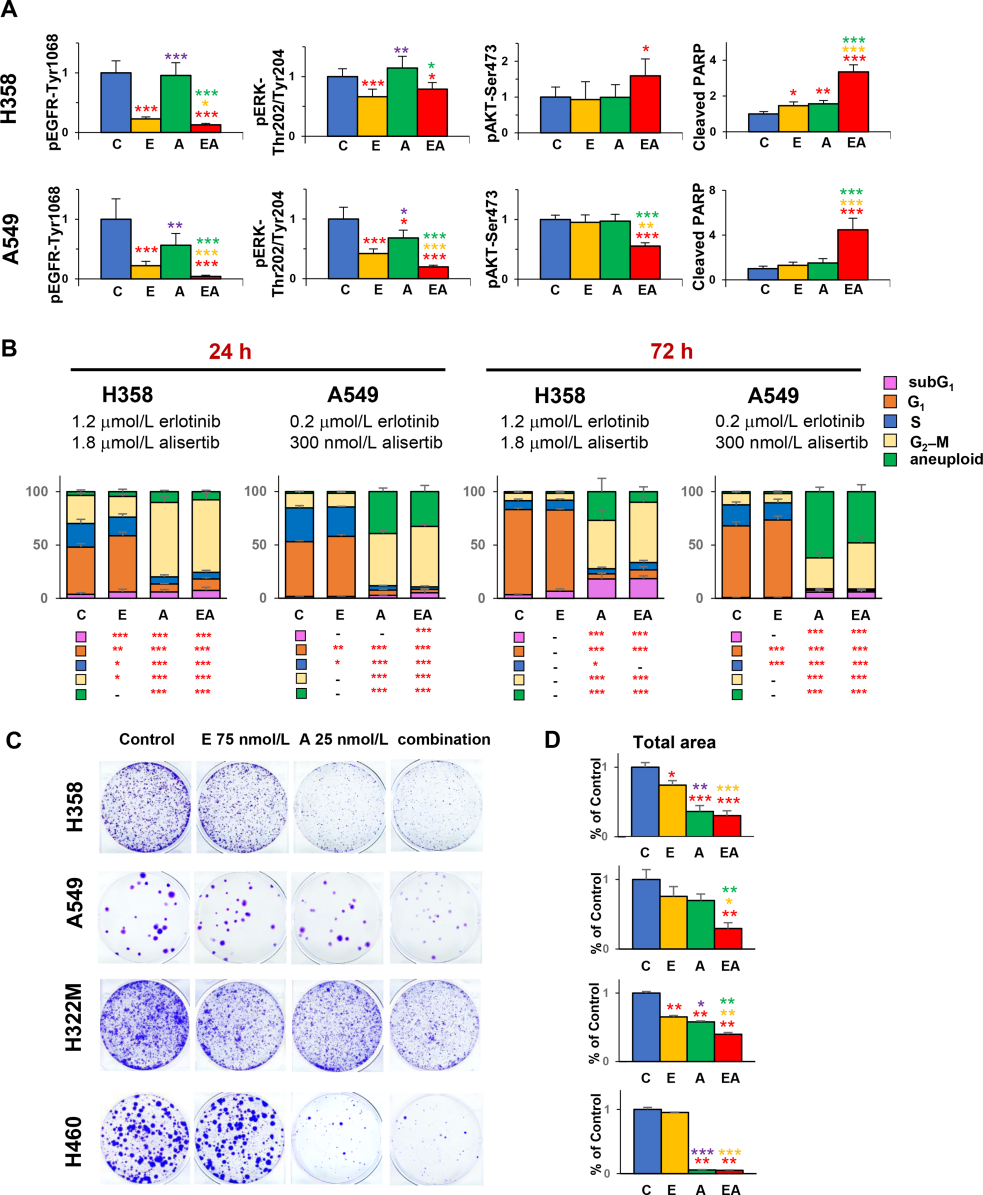
Signaling, cell cycle, and clonogenic analysis of combined erlotinib and alisertib in *KRAS^mut^* NSCLC cells. **A,** Quantification of Western analysis of drug-treated cells using indicated antibodies, based on five to eight replicates. Cells were treated for 24 hours with concentrations reflecting the IC_80_ value of erlotinib and/or alisertib (9 µmol/L erlotinib: 13 µmol/L alisertib, in A549 cells, or 2.8 µmol/L erlotinib: 4.2 µmol/L alisertib in H358 cells). Primary Western blot data are shown in Supplementary Fig. S1. **B,** FACS analysis of cell cycle in H358 and A549 cells treated for 24 or 72 hours with drug concentrations 0.2 µmol/L erlotinib: 300 nmol/L alisertib, in A549 cells, or 1.2 µmol/L erlotinib: 1.8 µmol/L alisertib in H358 cells; three to four biological replicates, data shown represent averaged values. Representative data from clonogenic assays (**C**) and quantitation of images from three biological replicates (**D**), for the indicated cell lines treated with negative control, 75 nmol/L erlotinib, 25 nmol/L alisertib, or the combination for 10 days. Statistical comparisons are to control (red), combination to erlotinib (orange) or alisertib (green), alisertib to erlotinib (purple). *, *P* < 0.05; **, *P* < 0.01; ***, *P* < 0.001. C, vehicle; E, erlotinib; A, alisertib; EA, erlotinib + alisertib.

After 24 hours drug treatment (Fig. 3A; Supplementary Fig. S1A and S1B), EGFR activity levels were reduced in erlotinib-treated cells, and to a significantly greater degree in cells treated with the erlotinib-alisertib combination. ERK activation was reduced in erlotinib and combination treated cells; however, the combination reduced activity levels beyond those achieved with erlotinib treatment alone only in A549 cells. The activity of the EGFR effector AKT was also significantly reduced in A549 cells by the combined action of two drugs, but not in H358 cells. Cell cycle analysis after 24 hours of drug treatment (Fig. 3B; Supplementary Fig. S1C) indicated that cells treated with erlotinib or vehicle were predominantly in G_1_, whereas cells treated with alisertib or the drug combination accumulated at G_2_–M, indicating efficient inhibition of AURKA. By 72 hours after drug addition, aneuploid (>4N) and sub-G_1_ populations increased for cells treated with either alisertib or the erlotinib+alisertib drug combination, associated with accumulating populations of nonviable cells. Notably, PARP cleavage, indicative of induction of apoptosis, was significantly elevated in cells treated with the drug combination versus either drug used as a single agent (Fig. 3A).

To further probe combination effect, we used a clonogenic assay to assess the effect of the alisertib and erlotinib combination in A549, H358, A322M, and H460 cells after 10 days continuous growth in the presence of drugs (erlotinib, 75 nmol/L; alisertib, 25 nmol/L; Fig. 3C and D). Both drugs were much more potent in clonogenic versus viability assays, and for two of the four cell lines, combination of the two drugs was more effective than use of single agents in reducing cell growth. In H460 cells, alisertib was extremely effective even at the low dose used, minimizing the contribution of additional erlotinib.

### Efficacy of Combined Erlotinib and Alisertib in EGFR-wt NSCLC Xenografts

**FIGURE 4:**
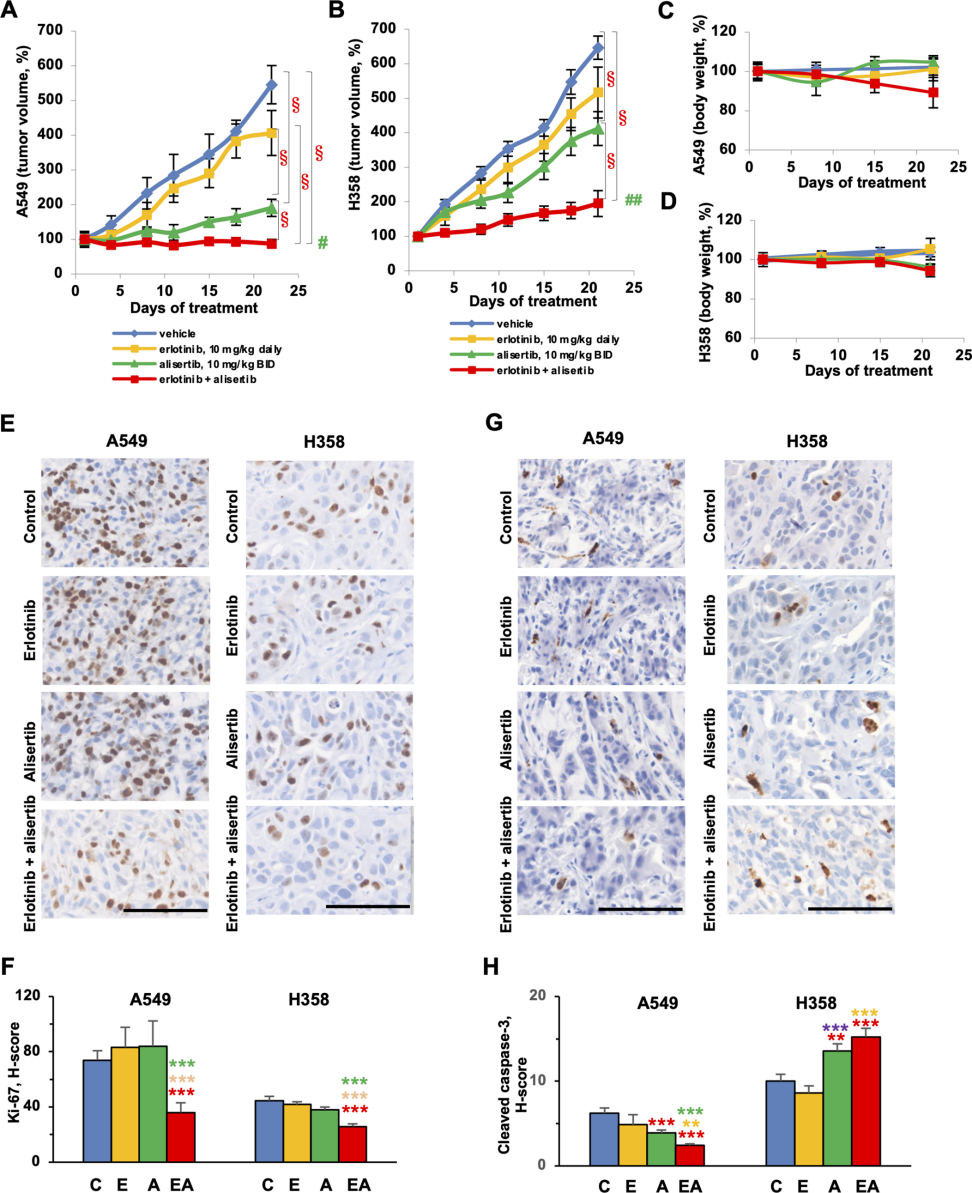
Combination of erlotinib and alisertib enhances suppression of *KRAS^mut^* NSCLC xenograft growth. A549 (**A**) and H358 (**B**) tumor xenografts, were treated with 10 mg/kg erlotinib, daily, and 10 mg/kg alisertib, twice daily, for 21 days. *n* = 5–8 for all groups. ^§^, *P* < 0.001; ^#^, additive effect; ^##^, synergistic effect (*P* = 0.011). Body weight of mice treated with erlotinib, alisertib or both for 21 days, from cohort shown in A (**C**) and B (**D**). Representative images and H-scores for Ki-67 (**E, F**) and cleaved caspase-3 (**G, H**) staining in A549 and H358 tumor xenografts treated with 10 mg/kg erlotinib, daily, 10 mg/kg alisertib, twice a day, or both for 21 days (mean ± SEM, *n* = 4–8). Scale bar, 100 µm. *, *P* < 0.05; **, *P* < 0.01; ***, *P* < 0.001, significantly different from comparison groups. Color of asterisks indicates comparison groups as follows: red, to control; orange, combination to erlotinib; green, combination to alisertib; purple, alisertib to erlotinib. C, vehicle; E, erlotinib; A, alisertib; EA, erlotinib + alisertib.

To assess the *in vivo* efficacy of combined erlotinib and alisertib in controlling the growth of KRAS^mut^ tumors, we established xenograft models from A549 and H358 NSCLC cells (Fig. 4). Commencing from when A549 xenografts reached 100–150 mm^3^, erlotinib was administered at a concentration of 10 mg/kg, and alisertib at 10 mg/kg twice a day (approximating the drug ration of 1:1.5 identified *in vitro*) for 3 weeks. Under these conditions, alisertib reduced tumor growth by 65% absolute difference versus vehicle-treated controls after 3 weeks in the A549 model. Conversely, single agent erlotinib did not significantly reduce growth in A549 xenografts. However, addition of erlotinib to alisertib completely eliminated tumor growth, with a much greater effect than alisertib alone (*P* < 0.001; Fig. 4A).

In the H358 tumor model (Fig. 4B), single agent erlotinib reduced tumor growth by 20% (not significant), and single agent alisertib reduced growth by 36%. However, the alisertib-erlotinib combination was extremely potent, reducing tumor growth by 70% absolute difference versus vehicle-treated controls (*P* < 0.001), and significantly versus either single agent (53% vs. alisertib, 62% vs. erlotinib). With both models, drug combinations were well tolerated, with no significant weight loss observed over the time of the experiment (Fig. 4C and D).

IHC staining with Ki-67 to detect proliferating cells in tumors collected following 3 weeks of drug treatment (Fig. 4E and F) showed marked reduction of cycling cells treated with the drug combination versus either drug used as a single agent, particularly in the A549 cells. Interestingly, no significant reduction was seen with alisertib-treated cells, even though these tumors had significant growth inhibition (Fig. 4A and B); we note, given the role of Ki-67 as a component of mitotic chromatin (40), it may underreport cell cycle arrest not occurring in early G_1_/G_0_. IHC analysis for cleaved caspase-3 in the same A549 tumors showed a reduction in alisertib- or alisertib+erlotinib treatment groups, whereas the opposite was observed in H358 xenografts (Fig. 4G and H). These data implied the combination inhibitory activity of alisertib+erlotinib was to control proliferation in A549 tumors, but both reduced proliferation and induced cell death in H358 tumors.

### Efficacy of Erlotinib and Alisertib in Repressing Proliferative Signaling in KRAS^mut^ Xenografts

**FIGURE 5:**
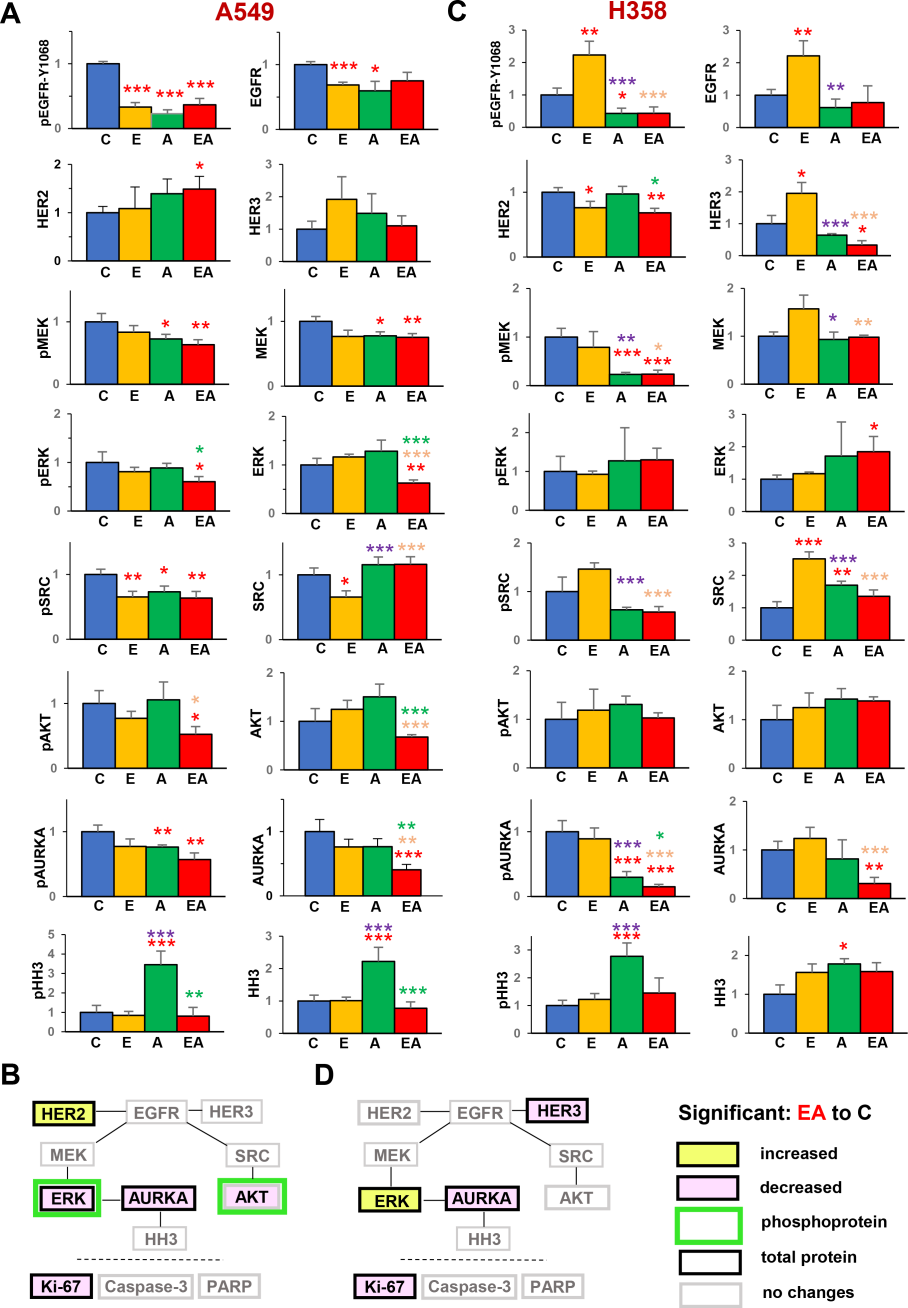
Signaling patterns in *KRAS^mut^* NSCLC xenografts after extended treatment with erlotinib and alisertib. Quantification of Western blot images for A549 tumors (**A**), with summary of analytes for which the result with the erlotinib+alisertib combination but not with any single drug differed significantly from the vehicle-treated control (**B**); data are presented as mean ± SEM, *n* = 4–8 mice. In A549, phospho-AURKA activity was determined by an MSD kit and normalized to the protein content; total AURKA was assessed by Western blotting. In H358, both AURKA forms were measured by Western blotting. Results as for A and B, but showing quantified data (**C**) and summary analysis (**D**) for H358 tumors. Primary Western blot data for both tumor models are shown in Supplementary Figs. S2 and S3. *, Significantly different from corresponding groups with *P* < 0.05; **, *P* < 0.01; or ***, *P* < 0.001. Color of asterisks indicates comparison groups as follows: red, to control; orange, combination to erlotinib; green, combination to alisertib, purple, alisertib to erlotinib. C, vehicle; E, erlotinib; A, alisertib; EA, erlotinib + alisertib.

To better characterize sustained signaling consequences of 3 weeks of drug treatment, Western blot analysis of tumor lysates was used to evaluate expression or activation of a panel of proteins relevant to EGFR and AURKA function in tumors collected from treated mice at the end of the experiment (Fig. 5; Supplementary Figs. S2 and S3). In both A549 and H358 xenograft tumors (Fig. 5A and C; Supplementary Figs. S2 and S3), erlotinib and alisertib used in combination inhibited EGFR phosphorylation at Tyr^1068^ by more than 60%, and modestly reduced the expression of total EGFR. AURKA activation was also inhibited by the drug combination, accompanied by reduced levels of the total protein; AURKA degradation has previously been shown to be enhanced by inhibition of autophosphorylation activity (41, 42). Interestingly, accumulation of phosphorylated histone H3, indicative of double stranded DNA breaks and accumulation in mitosis, was elevated by inhibition of AURKA, but not by the drug combination. These data indicated that the drugs remained active and inhibited their targets after 3 weeks.

Downstream of the drug targets, results were nonidentical between the two cell lines. In A549 cells, expression of the EGFR family member HER2 increased in cells treated with the drug combination (Fig. 5A and B). Among the effectors of EGFR, levels of active MEK, ERK, and AKT were more significantly reduced by the drug combination than by either single agent; however, this decrease in kinase activation was associated with a reduction in the total levels of the proteins. The kinase SRC interacts and cross-signals with both EGFR and AURKA (43–45); SRC activation was comparably inhibited by erlotinib and alisertib, although the combination did not result in greater reduction of SRC activity than either single agent.

The pattern of signaling changes in H358 xenograft tumors differed in some key respects from that observed in the A549 model (Fig. 5C and D; Supplementary Fig. S3). Most significant was the lack of erlotinib-induced inhibition of EGFR phosphorylation; rather, levels of ph^Y1068^-EGFR were elevated in erlotinib-treated tumors, although they were significantly depressed in tumors treated with alisertib or the drug combination. This may reflect the fact that total expression of EGFR and its family member HER3 protein levels were both enhanced by single agent erlotinib treatment, although reduced by the erlotinib-alisertib combination. Importantly, activation of the downstream EGFR effectors, ERK and AKT, levels were not reduced by any of the drugs tested, further suggesting compensatory activation mechanisms of EGFR effectors after several weeks of sustained treatment.

### Single Treatment with Erlotinib and Alisertib *In Vivo* Reveals Early Signaling Events in A549 and H358 Xenografts

**FIGURE 6:**
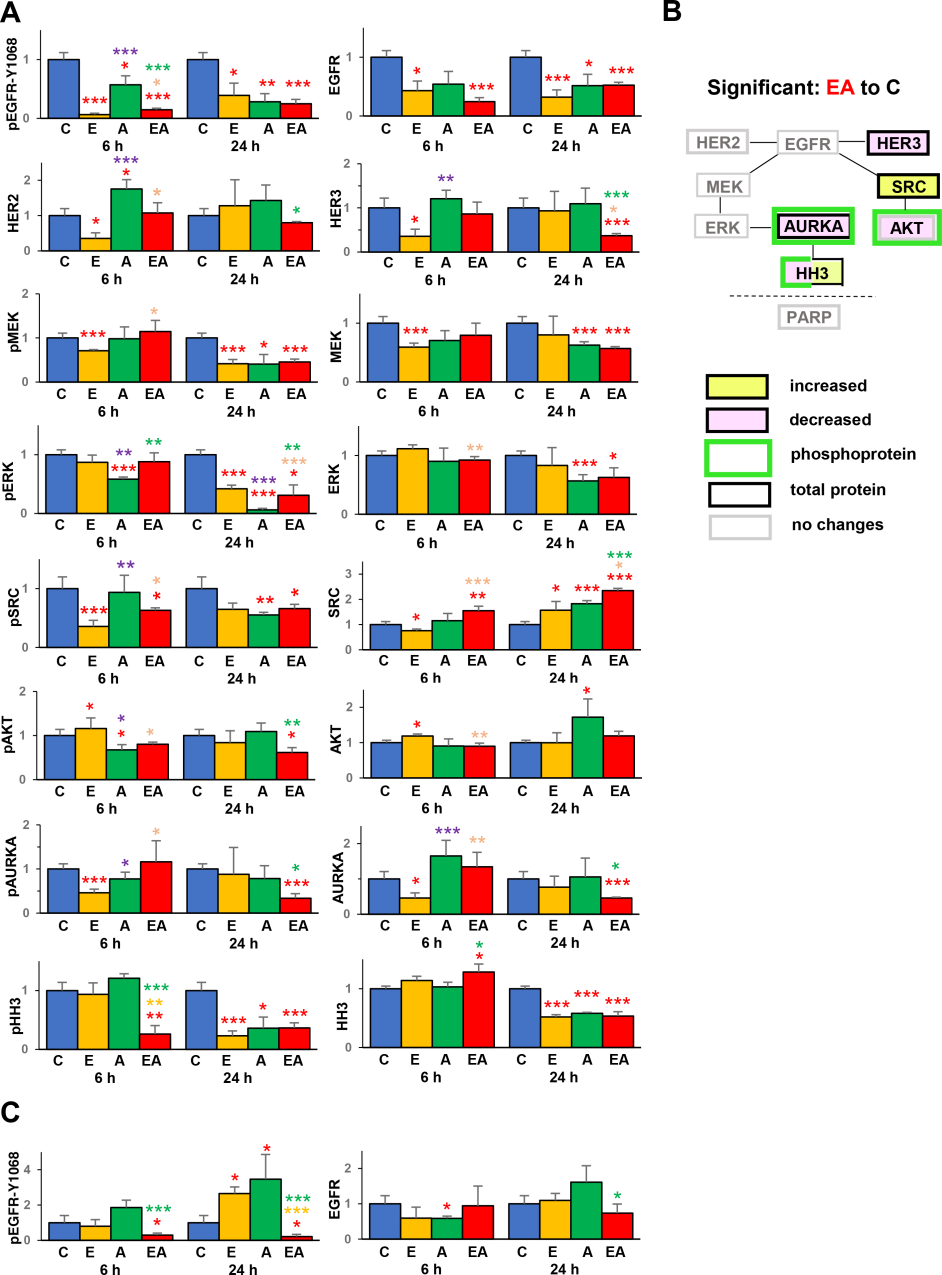
Signaling patterns in *KRAS^mut^* NSCLC xenografts 6 or 24 hours after treatment with erlotinib or/and alisertib. Quantification of Western blot images for A549 (**A**) xenograft tumors. Data are presented as mean ± SEM, *n* = 3–6 mice. **B,** Summary for A549 xenografts shows significant changes in protein levels and activation after erlotinib+alisertib treatment, in reference to vehicle treated control group. **C,** Quantification of Western blots for H358 xenograft tumors. Primary Western blot data are shown in Supplementary Figs. S4 and S5. *, Significantly different from corresponding groups with *P* < 0.05; **, *P* < 0.01; or ***, *P* < 0.001. Color of asterisks indicates comparison groups as follows: red, to control; orange, combination to erlotinib; green, combination to alisertib, purple, alisertib to erlotinib. C, vehicle; E, erlotinib; A, alisertib; EA, erlotinib + alisertib.

To better understand the direct effect of AURKA and EGFR inhibition on KRAS-mutated tumors prior to the selection of compensatory signaling changes, we performed additional xenograft studies with the A549 and H358 models. Mice bearing xenografts were given a single dose of erlotinib and two doses of alisertib, and tumors collected after 6 or 24 hours for preparation of lysates (Fig. 6; Supplementary Figs. S4 and S5).

In A549 xenografts (Fig. 6A and B), a single dose of erlotinib inhibited EGFR phosphorylation, as well as expression of total EGFR, HER2, and HER3 by 6 hours posttreatment. Both active (ph^Ser217/221^MEK and ph^Tyr418^SRC) and total MEK and SRC were also suppressed by erlotinib at this time point while active ph^Ser473^-AKT and total AKT were modestly increased. By 24 hours posttreatment, only ph^Tyr1068^- and total EGFR and ph^Ser217/221^-MEK remained suppressed. Alisertib alone reduced active EGFR, ERK, and AKT as early as 6 hours posttreatment and also active MEK and SRC by 24 hours after treatment. Inhibition of AURKA activity by alisertib was less marked, but the effect of the drug combination was significant by 24 hours after treatment for both active and total forms. Both alisertib and erlotinib significantly reduced ph^Thr202/Tyr204^-ERK within 24 hours of drug treatment, in concord with the reduced proliferation of cells treated with these drugs.

For the H358 xenografts, ability to perform analysis was limited by the facts that tumors in this model were highly necrotic, and the tumor volumes were much smaller compared with the tumors that grew for 3 weeks of treatment, providing sufficient material only to analyze EGFR. On the basis of this analysis (Fig. 6B, Supplementary Fig. S5), erlotinib used as a single agent was not sufficient to reduce EGFR activation at 6 and 24 hours postadministration, whereas the erlotinib-alisertib combination produced highly significant reduction.

## Discussion

Several conclusions can be drawn from the data in this study. First, analysis of large public datasets indicates that levels of AURKA are comparable or higher in *KRAS^mut^* versus *EGFR^mut^* NSCLC, with highest levels in tumors with mutated *TP53*; and levels of EGFR that are appreciable although lower are expressed in *KRAS^mut^* NSCLC tumors. Second, *KRAS^mut^* NSCLC cell models display a range of sensitivity to EGFR and AURKA inhibition; however, the degree of sensitivity *in vitro* does not correspond directly to expression of the EGFR or AURKA protein, or *TP53* mutation status. Third, the combination of alisertib and erlotinib is synergistic at multiple ratios in two *KRAS^mut^* cell line models *in vitro*, increasing aneuploidy and cell death, and is also effective at controlling xenograft growth of these models *in vivo*. Interestingly, the *in vivo* growth control can be linked to reduced cell division (A549 cells) or increased cell death (H358 cells) in different NSCLC models. Fourth, the alisertib-erlotinib combination is more effective than single agents in reducing levels of activated EGFR and its effectors, even in a genetic background lacking an EGFR driver mutation.

Although EGFR activates numerous downstream signaling effectors, including FAK, SRC, PLCγ, STATs, and others (46, 47), RAS has often been proposed to represent the most important mediator of EGFR biological activity. This interpretation has been supported by clinical findings in some tumor settings. For example, in colorectal cancers overexpressing EGFR, the presence of a strongly activating KRAS mutation eliminates the effectiveness of EGFR-targeting antibodies, even in tumors with highly overexpressed EGFR (48, 49). However, in EGFR-dependent NSCLC, where the oncogenic driver is more typically EGFR bearing a catalytically activating mutation rather than overexpression of EGFR, resistance to EGFR-targeting kinase inhibitors is very rarely associated with mutations activating RAS; rather, resistance usually is associated with additional mutations in EGFR, overexpression of other receptor tyrosine kinases (the RTKs ERBB2 and c-MET), mutations in PI3K or BRAF, or histologic transition (4, 50). This pattern of resistance suggests that activation of KRAS and of EGFR is not equivalent. Another related study found that treatment with a KRAS^G12C^ inhibitor caused cells to initially assume a drug-induced quiescent state; subsequently, upregulation of the EGFR ligand HB-EGF caused autocrine EGFR activation, promoting the new synthesis of KRAS^G12C^ (51).

The predominant AURKA functions in untransformed cells involve physical association with, and regulation of, ciliary basal bodies, centrosomes, and the mitotic apparatus (23). However, it has become clear that the profile of AURKA targets is greatly expanded in transformed cells overexpressing AURKA, due in part to the expanded localization of the protein to include the cytoplasm and the nucleus, as well as elevated activity of AURKA in additional phases of cell cycle (52). AURKA contributes to resistance to clinically valuable inhibitors of diverse regulatory kinases that are closely linked to EGFR and RAS signaling, including SRC (43), PI3K (53), mTOR (54), RAF (55), and EGFR (27, 56, 57), among others. Relevant to this study, treatment of KRAS^G12C^ lung cancer cells with sotorasib has been shown to induce the increased expression of AURKA, which then directly interacted with KRAS^G12C^, locking KRAS into an active, KRAS^G12C^ inhibitor–insensitive state (51). *In vivo*, tumors treated for a short time (6 or 24 hours, Fig. 6) with the drug combination had reduced activation of numerous EGFR effectors, in accord with its reported activity. Direct AURKA interaction and phosphorylation of some of these effectors has been reported in some contexts [e.g., with SRC (43)], and disruption of one or more likely several such interactions may contribute to the combination effect observed in inhibiting tumor growth.

Our data suggest that it may be useful to evaluate the use of EGFR and AURKA inhibitors in a broader group of patients with NSCLC. NSCLC and other lung cancers remain the most common avoidable cancers in the United States and globally (58). Although EGFR-mutated NSCLC is typically not associated with smoking and has a lower mutation burden and better prognosis, KRAS-mutated NSCLC has a much higher level of DNA damage, and patients often suffer from smoking-associated comorbidities including chronic obstructive pulmonary disease (59), hypertension, and other metabolic disorders. AURKA is considered a promising target in other smoking-related cancers (60, 61). Interestingly, besides the regulating directly relevant signaling targets in KRAS-mutated lung cancers, additional functions of AURKA continue to be identified, and include roles in DNA replication and repair (62, 63), hypoxia signaling (64), and other metabolic processes (65, 66); it is likely that these roles also contribute to supporting malignant growth, and merit further investigation. On the basis of our data, gross levels of total EGFR or AURKA are not sufficient to provide biomarkers for which tumors are likely to respond. This likely reflects the considerable complexity of factors modulating EGFR and AURKA signaling pathways (4, 23, 67), as reflected in the nonequivalent activities of combining alisertib and erlotinib in A549 and H358 cells in this study. Our data suggest that identification of response biomarkers for these EGFR and AURKA inhibitors in the setting of NSCLC is merited.

## Supplementary Material

Supplementary Data List of antibodies

Supplementary Data Additional information for Fig.1

Figure S1 Primary images related to Fig.3

Figure S2 Primary images for Fig.5A

Figure S3 Primary images for Fig.5C

Figure S4 Primary images for Fig.6A

Figure S5 Primary images for Fig.6C
