# Supplementary material for: Synergy of EGFR and AURKA Inhibitors in KRAS-mutated Non–small Cell Lung Cancers
Source: Cancer Res Commun. 2024 May 8;4(5):1227–39. doi: 10.1158/2767-9764.CRC-23-0482 (PMC11078142; doi:10.1158/2767-9764.CRC-23-0482)
Supplement: Supplementary Data — List of antibodies [file crc-23-0482-s01.pdf]

Supplementary Table S1
List of antibodies

Antibodies	Company	Cat#	Dilution
**β-Actin** Rabbit Antibody RRID:AB_330288	Cell Signaling Technology	4967	1:1000
**Akt** (pan) (40D4) Mouse mAb RRID:AB_1147620	Cell Signaling Technology	2920	1:1000
**Phospho-Akt (Ser473)** (D9E) XP® Rabbit mAb; RRID:AB_2315049	Cell Signaling Technology	4060	1:1000
**Aurora A/AIK** Antibody RRID:AB_2061342	Cell Signaling Technology	3092	1:1000
**Phospho-Aurora A** (Thr288)/Aurora B (Thr232)/Aurora C (Thr198) (D13A11) XP® Rabbit mAb; RRID:AB_2061631	Cell Signaling Technology	2914	1:1000
Purified Mouse Anti-**EGF Receptor** Antibody; RRID:AB_2096701	BD Biosciences	610017	1:1000
**Phospho-EGFR (Tyr1068)** (D7A5) XP® Rabbit mAb; RRID:AB_2096270	Cell Signaling Technology	3777	1:1000
**GAPDH** Mouse mAb; RRID:AB_1078991	Sigma-Aldrich	G8795	1:20000
**HER2**/ErbB2 (29D8) Rabbit mAb RRID:AB_10692490	Cell Signaling Technology	2165	1:1000
**HER3**/ErbB3 (1B2E) Rabbit mAb RRID:AB_10691324	Cell Signaling Technology	4754	1:1000
**Histone H3** Antibody RRID:AB_331563	Cell Signaling Technology	9715	1:2000
**Phospho-Histone H3 (Ser10)** (D2C8) XP® Rabbit mAb RRID:AB_1549592	Cell Signaling Technology	3377	1:1000
**MEK1/2** (L38C12) Mouse mAb RRID:AB_10695868	Cell Signaling Technology	4694	1:1000
**Phospho-MEK1/2 (Ser217/221)** (41G9) Rabbit mAb; RRID:AB_2138017	Cell Signaling Technology	9154	1:1000
**p44/42 MAPK (Erk1/2)** (L34F12) Mouse mAb; RRID:AB_390780	Cell Signaling Technology	4696	1:1000
**Phospho-p44/42 MAPK (Erk1/2) (Thr202/Tyr204)** Antibody RRID:AB_331646	Cell Signaling Technology	9101	1:1000
**PARP** Antibody; RRID:AB_2160739	Cell Signaling Technology	9542	1:1000
**Src** (L4A1) Mouse mAb; RRID:AB_10691385	Cell Signaling Technology	2110	1:1000
**phospho-Src (Tyr418)** Rabbit Antibody RRID:AB_304652	Abcam	ab4816	1:1000
**Vimentin** (D21H3) XP® Rabbit mAb RRID:AB_10695459	Cell Signaling Technology	5741	1:1000
Antibodies for IHC:			
**Cleaved Caspase-3** (Asp175) Antibody RRID:AB_2341188	Cell Signaling Technology	9661	1:400
**Ki-67** (MIB-1) Mouse mAb RRID:AB_2142367	Dako (now Agilent)	M7240	1:100
