# Supplementary material for: Synergy of EGFR and AURKA Inhibitors in KRAS-mutated Non–small Cell Lung Cancers
Source: Cancer Res Commun. 2024 May 8;4(5):1227–39. doi: 10.1158/2767-9764.CRC-23-0482 (PMC11078142; doi:10.1158/2767-9764.CRC-23-0482)
Supplement: Supplementary Data — Additional information for Fig.1 [file crc-23-0482-s02.pdf]

Supplementary Table S2. Related to Figure 1. Detailed statistical data for comparison groups in Figure 1, reporting sample size, assessment of data normality (using Anderson-Darling test), medians, variances, means, and the first (Q1) and third (Q3) quartiles. Each of these values is separated by a semi-colon and corresponds to each violin plot on Fig 1, moving from left to right. The table also includes the results of the Kolmogorov-Smirnov and Wilcoxon tests, represented as p-values, which indicate the statistical significance of difference between the compared groups. In the case of two groups, a single p-value represents the comparison between them. For four groups, two p-values are provided: one for the comparison between the first and second group (TP53 MUT vs TP53 WT), and another for the comparison between the third and fourth group (TP53 MUT vs TP53 WT). Conclusions are based on the results of the Kolmogorov-Smirnov tests.

Fig.1	Sample sizes	Data normality (Anderson-Darling test p-values)	Medians	Variances	Means	Q1	Q3	Kolmogorov-Smirnov test (p-values)	Wilcoxon test (p-values)
A	207; 205	8.15e-05; 0.5164	0.53; -0.14	1; 0.64	0.6; -0.17	0.04; -0.8	1.2; 0.37	1.281E-13	2.2E-16
B	167; 172	9.424E-05; 0.0008	0.48; -0.3	2.35; 0.57	-0.82; -0.18	-0.16; 1.4	-0.66; 0.19	3.944E-05	1.317E-06
C	207; 205	0.16; 0.32	-0.16; 0.18	0.9; 1	-0.07; 0.08	-0.79; 0.58	-0.62; 0.79	0.007607	0.05778
D	-	-	-	-	-	-		-	-
E	110; 97; 80; 125	0.57; 0.09; 0.56; 0.05	0.14; -0.49; 0.43; -0.17	0.88; 0.71; 0.73; 1	0.22; -0.4; 0.46; -0.17	-0.34; -1; -0.04; -1	0.86; 0.08; 0.96; 0.6	1.48E-05; 0.0001	5.254E-07; 3.119e-05
F	110; 97; 80; 125	0.005; 0.004; 0.05; 0.6;	0.72; 0.37; 0.05; -0.31	1.4; 0.67; 0.79; 0.51	0.75; 0.43, 0.015; -0.3	0.2; 0.02; -0.37; -0.85	1.4; 0.8; 0.6; 0.24	0.001; 0.005	0.003; 0.001
