# Supplementary material for: Synergy of EGFR and AURKA Inhibitors in KRAS-mutated Non–small Cell Lung Cancers
Source: Cancer Res Commun. 2024 May 8;4(5):1227–39. doi: 10.1158/2767-9764.CRC-23-0482 (PMC11078142; doi:10.1158/2767-9764.CRC-23-0482)
Supplement: Figure S1 — Primary images related to Fig.3 [file crc-23-0482-s03.pptx]

## Slide 1
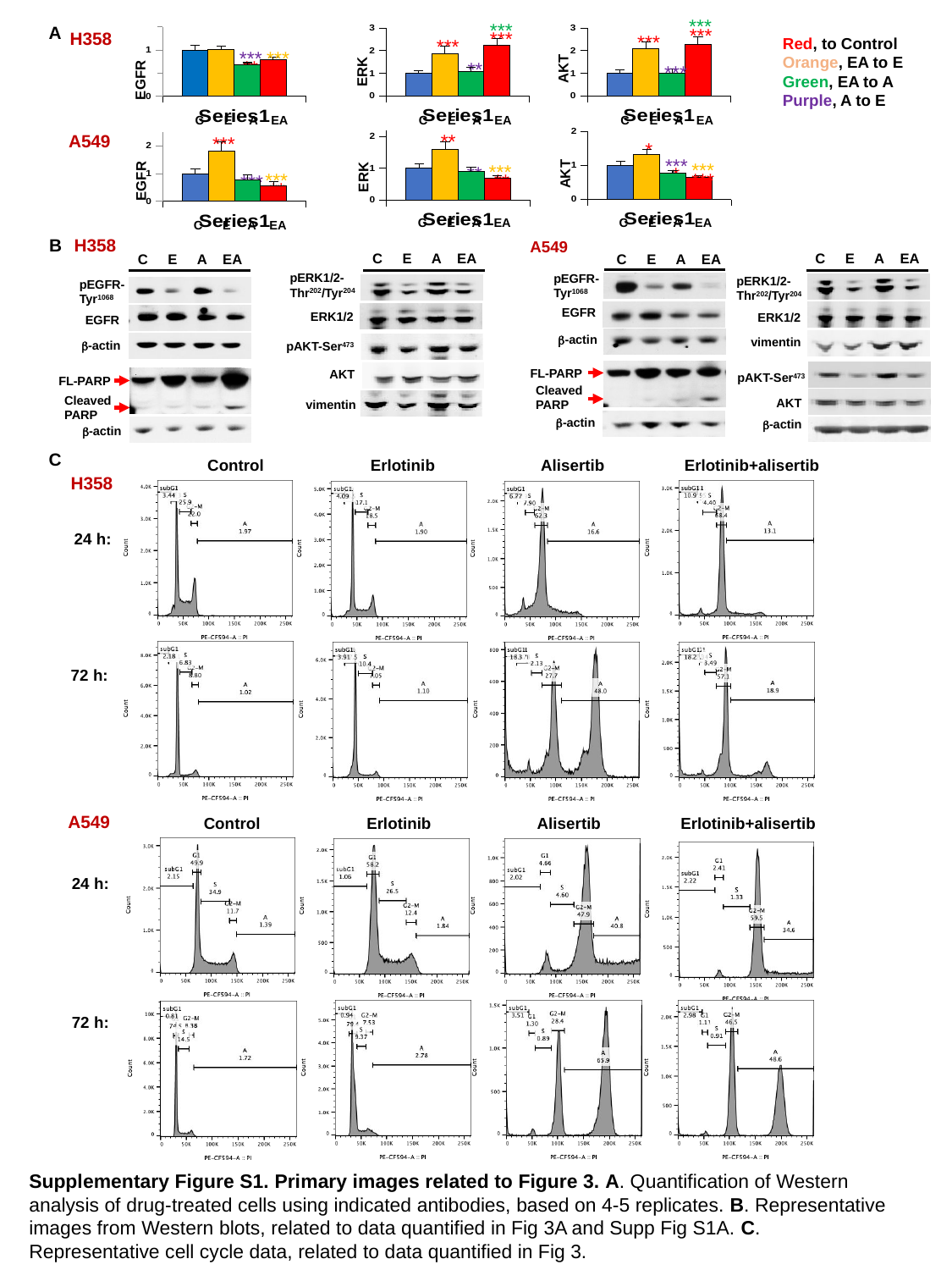

***
***
### Chart
| Category | Control | Erlotinib | Alisertib | Combo |
|---|---|---|---|---|
| | 1.0 | 2.1045 | 1.0026 | 2.2891 |***
AKT
***
C E A EA
***
***
### Chart
| Category | Control | Erlotinib | Alisertib | Combo |
|---|---|---|---|---|
| | 1.0 | 1.8729 | 1.0986 | 2.2387 |***
**
ERK
C E A EA
H358
### Chart
| Category | Control | Erlotinib | Alisertib | Combo |
|---|---|---|---|---|
| | 1.0 | 1.017 | 0.6737 | 0.8003 |***
***
**
EGFR
C E A EA
Red, to Control
Orange, EA to E
Green, EA to A
Purple, A to E
**
### Chart
| Category | Control | Erlotinib | Alisertib | Combo |
|---|---|---|---|---|
| | 1.0 | 1.589 | 0.9152 | 0.698 |***
**
ERK
**
C E A EA
A549
***
### Chart
| Category | Control | Erlotinib | Alisertib | Combo |
|---|---|---|---|---|
| | 1.0 | 1.8143 | 0.786 | 0.5626 |***
***
EGFR
**
C E A EA
### Chart
| Category | Control | Erlotinib | Alisertib | Combo |
|---|---|---|---|---|
| | 1.0 | 1.3229 | 0.7621 | 0.6468 |*
***
***
AKT
*
***
C E A EA
A
B
H358
A549
C E A EA
pERK1/2-
Thr202/Tyr204
ERK1/2
vimentin
pAKT-Ser473
AKT
b-actin
C E A EA
pERK1/2-
Thr202/Tyr204
ERK1/2
pAKT-Ser473
AKT
vimentin
C E A EA
pEGFR-
Tyr1068
EGFR
b-actin
FL-PARP
Cleaved
PARP
b-actin
C E A EA
pEGFR-
Tyr1068
EGFR
b-actin
FL-PARP
Cleaved
PARP
b-actin
C
Control Erlotinib Alisertib Erlotinib+alisertib
H358
24 h:
72 h:
A549
Control Erlotinib Alisertib Erlotinib+alisertib
24 h:
72 h:
Supplementary Figure S1. Primary images related to Figure 3. A. Quantification of Western analysis of drug-treated cells using indicated antibodies, based on 4-5 replicates. B. Representative images from Western blots, related to data quantified in Fig 3A and Supp Fig S1A. C. Representative cell cycle data, related to data quantified in Fig 3.
