# Supplementary material for: Synergy of EGFR and AURKA Inhibitors in KRAS-mutated Non–small Cell Lung Cancers
Source: Cancer Res Commun. 2024 May 8;4(5):1227–39. doi: 10.1158/2767-9764.CRC-23-0482 (PMC11078142; doi:10.1158/2767-9764.CRC-23-0482)
Supplement: Figure S2 — Primary images for Fig.5A [file crc-23-0482-s04.pptx]

## Slide 1
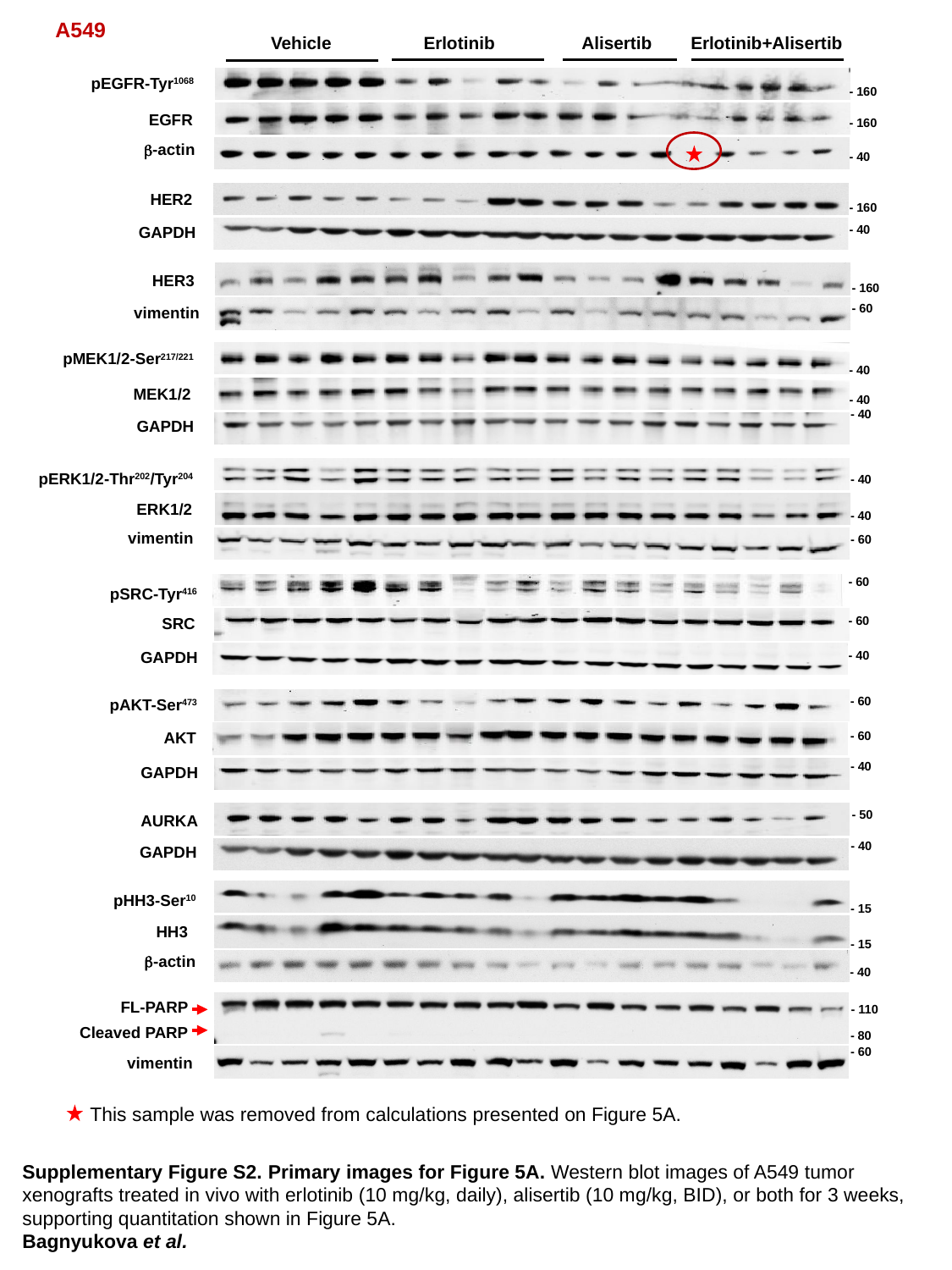

A549
 Vehicle Erlotinib Alisertib Erlotinib+Alisertib
pEGFR-Tyr1068
- 160
EGFR
- 160
b-actin
- 40
HER2
- 160
- 40
GAPDH
HER3
- 160
- 60
vimentin
pMEK1/2-Ser217/221
- 40
MEK1/2
- 40
- 40
GAPDH
pERK1/2-Thr202/Tyr204
- 40
ERK1/2
- 40
vimentin
- 60
- 60
pSRC-Tyr416
- 60
SRC
GAPDH
- 40
- 60
pAKT-Ser473
AKT
- 60
- 40
GAPDH
- 50
AURKA
- 40
GAPDH
pHH3-Ser10
- 15
HH3
- 15
b-actin
- 40
FL-PARP
- 110
Cleaved PARP
- 80
- 60
vimentin
This sample was removed from calculations presented on Figure 5A.
Supplementary Figure S2. Primary images for Figure 5A. Western blot images of A549 tumor xenografts treated in vivo with erlotinib (10 mg/kg, daily), alisertib (10 mg/kg, BID), or both for 3 weeks, supporting quantitation shown in Figure 5A.
Bagnyukova et al.
