# Supplementary material for: Synergy of EGFR and AURKA Inhibitors in KRAS-mutated Non–small Cell Lung Cancers
Source: Cancer Res Commun. 2024 May 8;4(5):1227–39. doi: 10.1158/2767-9764.CRC-23-0482 (PMC11078142; doi:10.1158/2767-9764.CRC-23-0482)
Supplement: Figure S3 — Primary images for Fig.5C [file crc-23-0482-s05.pptx]

## Slide 1
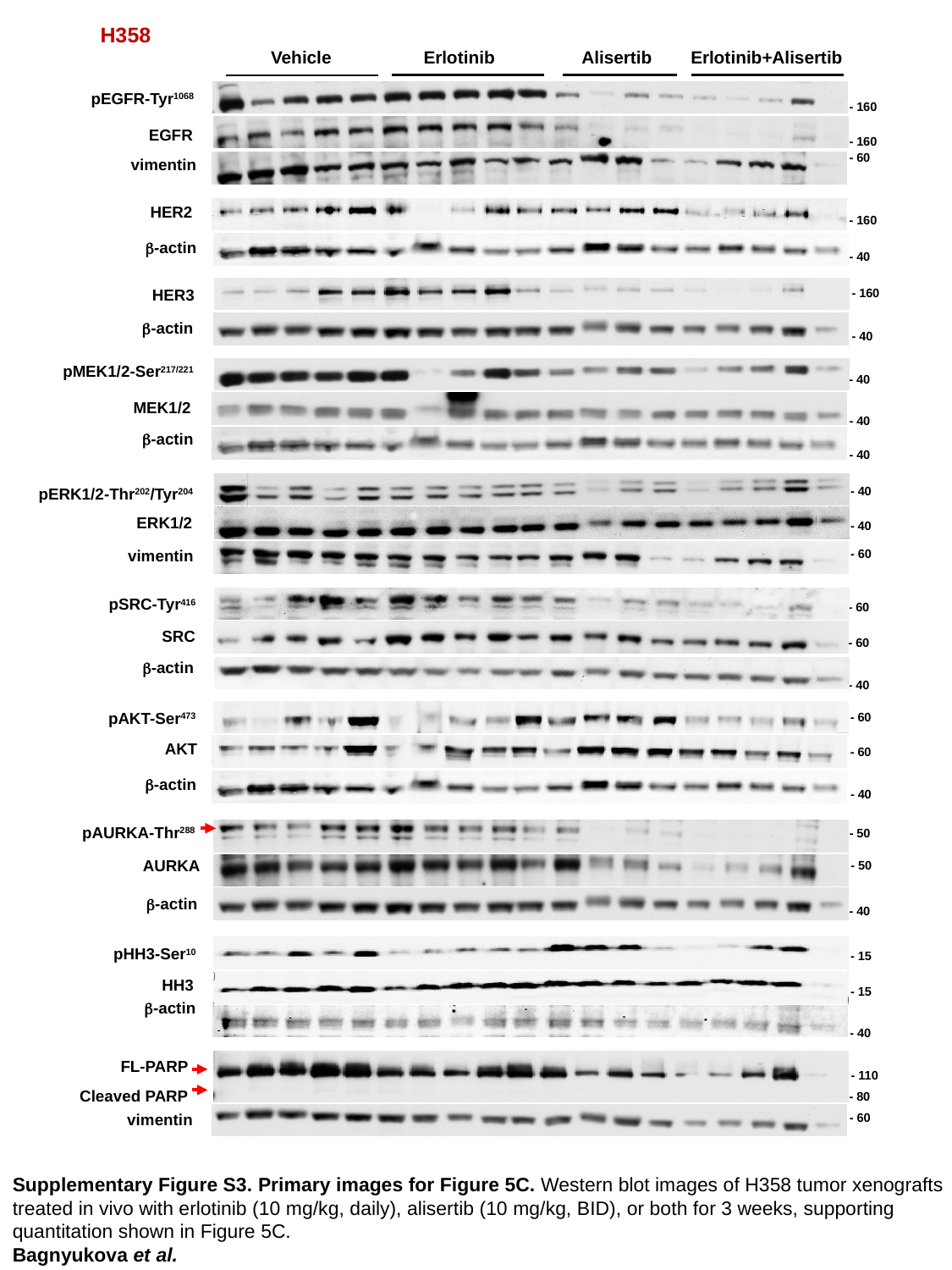

H358
 Vehicle Erlotinib Alisertib Erlotinib+Alisertib
pEGFR-Tyr1068
- 160
EGFR
- 160
- 60
vimentin
HER2
- 160
b-actin
- 40
HER3
- 160
b-actin
- 40
pMEK1/2-Ser217/221
- 40
MEK1/2
- 40
b-actin
- 40
- 40
pERK1/2-Thr202/Tyr204
ERK1/2
- 40
vimentin
- 60
pSRC-Tyr416
- 60
SRC
- 60
b-actin
- 40
pAKT-Ser473
- 60
AKT
- 60
b-actin
- 40
pAURKA-Thr288
- 50
AURKA
- 50
b-actin
- 40
pHH3-Ser10
- 15
HH3
- 15
b-actin
- 40
FL-PARP
- 110
Cleaved PARP
- 80
- 60
vimentin
Supplementary Figure S3. Primary images for Figure 5C. Western blot images of H358 tumor xenografts treated in vivo with erlotinib (10 mg/kg, daily), alisertib (10 mg/kg, BID), or both for 3 weeks, supporting quantitation shown in Figure 5C.
Bagnyukova et al.
