# Supplementary material for: Synergy of EGFR and AURKA Inhibitors in KRAS-mutated Non–small Cell Lung Cancers
Source: Cancer Res Commun. 2024 May 8;4(5):1227–39. doi: 10.1158/2767-9764.CRC-23-0482 (PMC11078142; doi:10.1158/2767-9764.CRC-23-0482)
Supplement: Figure S4 — Primary images for Fig.6A [file crc-23-0482-s06.pptx]

## Slide 1
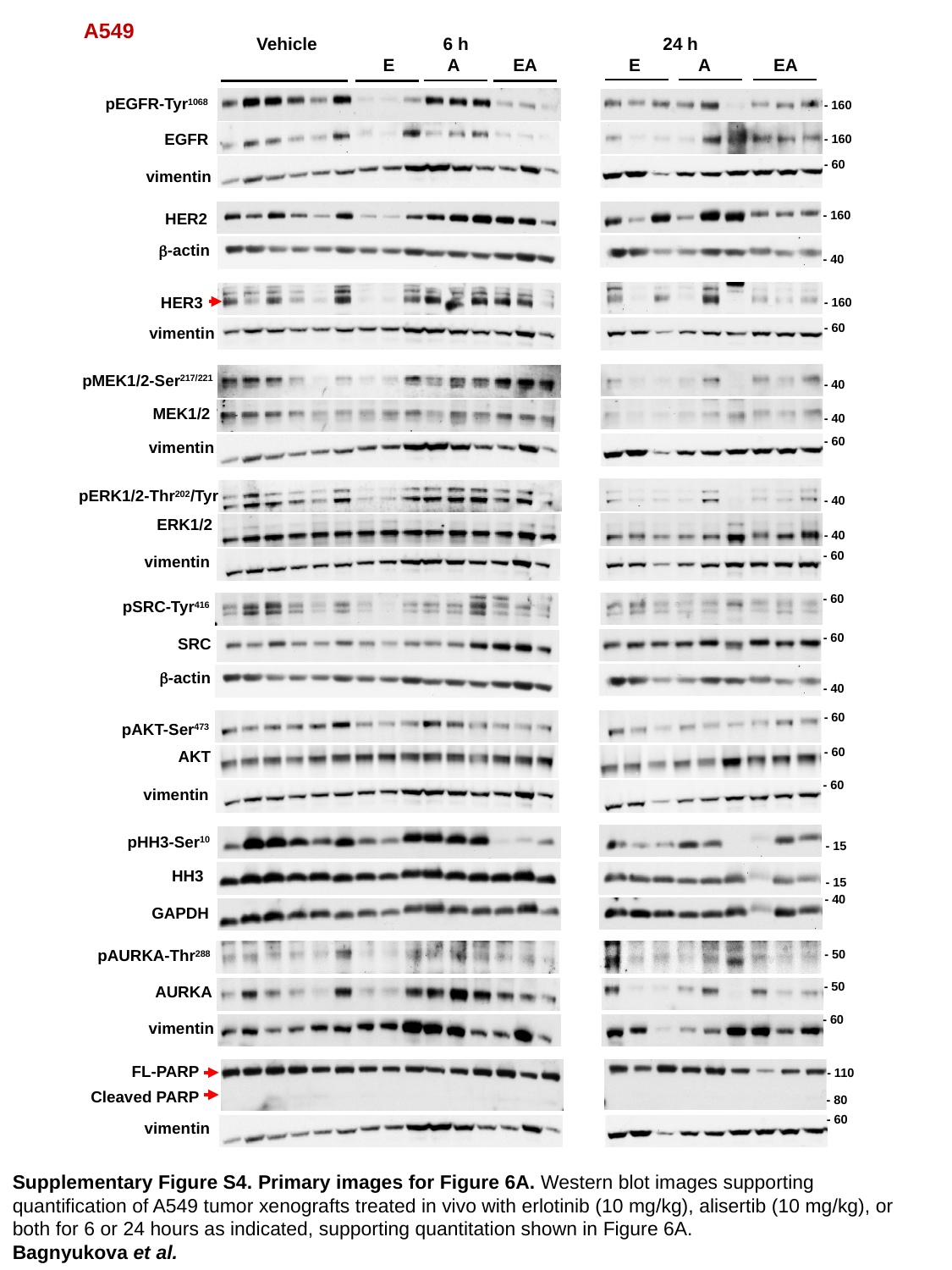

A549
 Vehicle 6 h 24 h
 E A EA E A EA
pEGFR-Tyr1068
- 160
EGFR
- 160
- 60
vimentin
- 160
HER2
b-actin
- 40
HER3
- 160
- 60
vimentin
pMEK1/2-Ser217/221
- 40
MEK1/2
- 40
- 60
vimentin
pERK1/2-Thr202/Tyr204
- 40
ERK1/2
- 40
- 60
vimentin
- 60
pSRC-Tyr416
- 60
SRC
b-actin
- 40
- 60
pAKT-Ser473
- 60
AKT
- 60
vimentin
pHH3-Ser10
- 15
HH3
- 15
- 40
GAPDH
pAURKA-Thr288
- 50
- 50
AURKA
- 60
vimentin
FL-PARP
- 110
Cleaved PARP
- 80
- 60
vimentin
Supplementary Figure S4. Primary images for Figure 6A. Western blot images supporting quantification of A549 tumor xenografts treated in vivo with erlotinib (10 mg/kg), alisertib (10 mg/kg), or both for 6 or 24 hours as indicated, supporting quantitation shown in Figure 6A.
Bagnyukova et al.
