# Supplementary material for: Synergy of EGFR and AURKA Inhibitors in KRAS-mutated Non–small Cell Lung Cancers
Source: Cancer Res Commun. 2024 May 8;4(5):1227–39. doi: 10.1158/2767-9764.CRC-23-0482 (PMC11078142; doi:10.1158/2767-9764.CRC-23-0482)
Supplement: Figure S5 — Primary images for Fig.6C [file crc-23-0482-s07.pptx]

## Slide 1
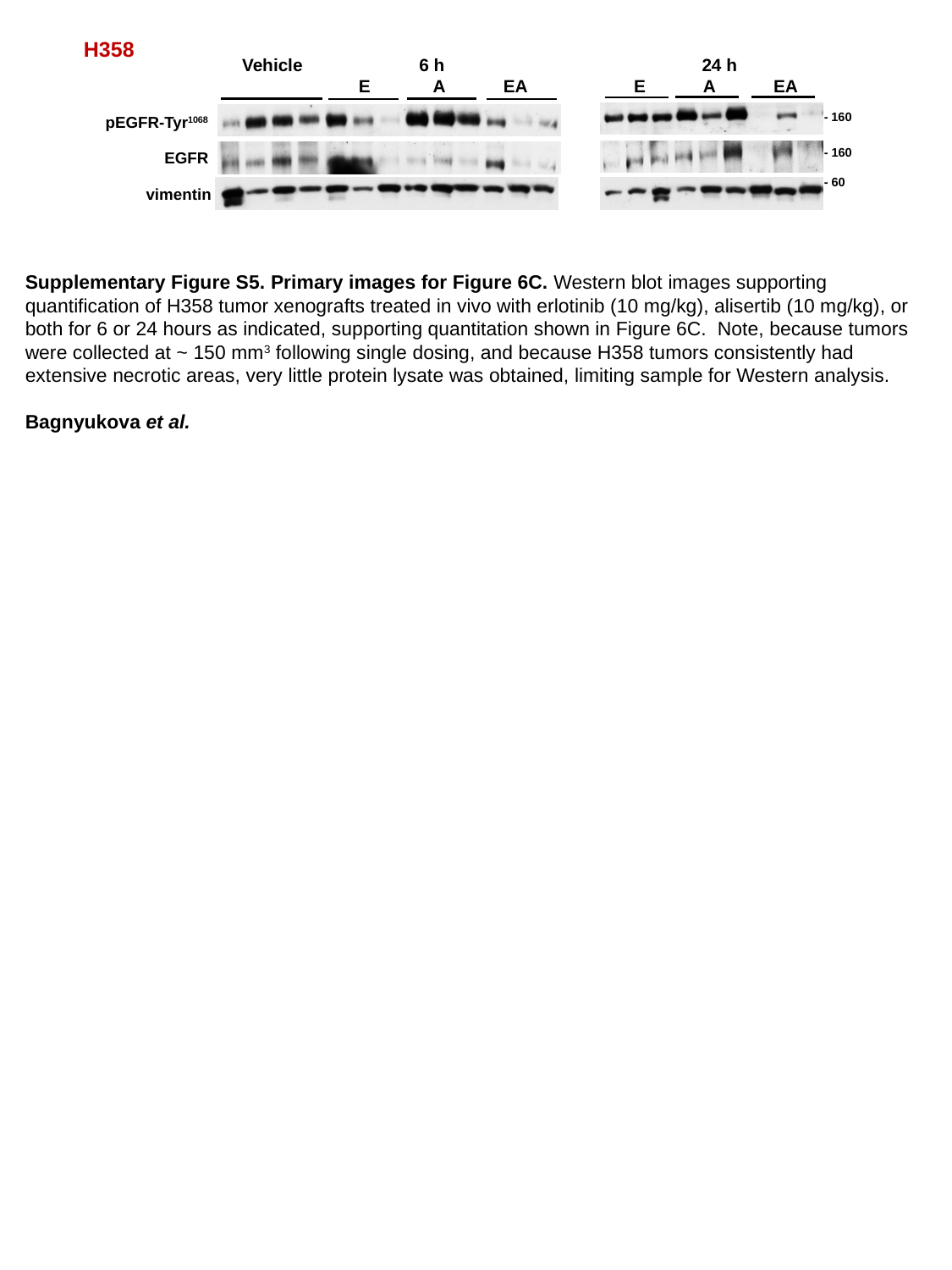

H358
 Vehicle 6 h 24 h
 E A EA E A EA
- 160
pEGFR-Tyr1068
- 160
EGFR
- 60
vimentin
Supplementary Figure S5. Primary images for Figure 6C. Western blot images supporting quantification of H358 tumor xenografts treated in vivo with erlotinib (10 mg/kg), alisertib (10 mg/kg), or both for 6 or 24 hours as indicated, supporting quantitation shown in Figure 6C. Note, because tumors were collected at ~ 150 mm3 following single dosing, and because H358 tumors consistently had extensive necrotic areas, very little protein lysate was obtained, limiting sample for Western analysis.
Bagnyukova et al.
